# Opportunistic computed tomography-based vertebral attenuation for osteoporosis screening

**DOI:** 10.1590/1806-9282.20260914

**Published:** 2026-10-23

**Authors:** Irfan Atik, Aibek Tashimbekov, Enes Gul, Seda Atik, Bulent Yildiz

**Affiliations:** 1 Sivas Cumhuriyet University, Faculty of Medicine, Department of Radiology – Sivas, Turkey.; 2 Erciyes University, Faculty of Medicine, Department of Radiology – Kayseri, Turkey.; 3 Sivas Cumhuriyet University, Faculty of Medicine, Department of Physical Medicine and Rehabilitation, Division of Rheumatology – Sivas, Turkey

**Keywords:** Biomarkers, Bone density, Computed tomography, Osteoporosis

## Abstract

**OBJECTIVE:**

The aim of this study was to evaluate opportunistic vertebral trabecular attenuation on routine unenhanced abdominal computed tomography for predicting osteoporosis and its relationship with dual-energy X-ray absorptiometry parameters.

**METHODS:**

This retrospective study inc luded 350 patients (mean age 68.5 years) who underwent both dual-energy X-ray absorptiometry and routine unenhanced abdominal computed tomography for non-oncologic clinical indications within a 3-month interval between 2022 and 2025. Patients were grouped into normal (n=119), osteopenia (n=117), and osteoporosis (n=114) based on L1–L4 T-scores. Two blinded radiologists measured Hounsfield Unit values using regions of interest on high-resolution 0.625-mm sagittal-reconstructed computed tomography images of the L1–L4 vertebral bodies. Interobserver agreement was assessed using intraclass correlation coefficients. Diagnostic performance was evaluated using receiver operating characteristic curve analysis.

**RESULTS:**

Lumbar Hounsfield Unit values decreased progressively from normal to osteoporosis groups (p<0.001). Mean L1–L4 attenuation values for normal, osteopenia, and osteoporosis groups were 148.99±5.54, 120.39±3.84, and 78.53±3.36 Hounsfield Unit, respectively (p<0.001). Interobserver reliability was excellent (intraclass correlation coefficient: 0.90–0.94). L1 Hounsfield Unit measurements showed the highest diagnostic performance for predicting osteoporosis (area under the curve: 0.913; 95%CI 0.870–0.955). An L1 cutoff of ≤95.50 Hounsfield Unit yielded 84.60% sensitivity and 86.40% specificity. Mean L1–L4 Hounsfield Unit also showed high accuracy (area under the curve: 0.881) at a ≤95.00 Hounsfield Unit cutoff (sensitivity: 80.80%, specificity: 77.30%).

**CONCLUSION:**

Opportunistic sagittal lumbar Hounsfield Unit measurements, particularly at L1, provide high diagnostic accuracy and reproducibility for identifying osteoporosis on routine abdominal computed tomography without requiring additional imaging or radiation exposure. This protocol may facilitate early opportunistic screening for osteoporosis and support timely referral for dual-energy X-ray absorptiometry assessment.

## INTRODUCTION

Osteoporosis (OP) is a common metabolic bone disease characterized by reduced bone mineral density (BMD) and deterioration of bone microarchitecture, resulting in an increased fracture risk^1^. Osteoporotic fractures are associated with substantial mortality, morbidity, and functional impairment, representing a major public health burden^2^. Despite its high prevalence, OP remains frequently undiagnosed in clinical practice, partly because it is often asymptomatic and requires dedicated diagnostic evaluation^3,4^. Opportunistic assessment of BMD from imaging studies performed for other clinical indications may facilitate the identification of at-risk individuals using existing diagnostic data.

Dual-energy X-ray absorptiometry (DXA) is currently the gold standard for OP diagnosis. However, DXA has several limitations. Degenerative changes and vascular calcifications, particularly in older adults, may lead to overestimation of lumbar spine BMD^5,6^. In addition, DXA requires dedicated examinations, resulting in additional healthcare utilization and potential loss of patients during screening and follow-up. Consequently, opportunistic vertebral density assessment using computed tomography (CT) examinations acquired for other indications has emerged as a practical alternative approach^7,8^.

Abdominal CT is frequently performed in routine clinical practice and provides valuable information regarding vertebral trabecular bone structure. Vertebral trabecular attenuation can be rapidly quantified in Hounsfield Units (HUs), providing an indirect measure of BMD. Previous studies have demonstrated significant correlations between lumbar vertebral HU values and DXA-derived T-scores^7,8^. Furthermore, CT-based measurements show high intra- and interobserver reproducibility and require no additional imaging^9,10^. Sagittal CT reconstructions also enable assessment of vertebral compression fractures^11^, while low vertebral trabecular attenuation values have been associated with prevalent and future major osteoporotic fractures^12,13^.

This study aimed to evaluate the diagnostic value of opportunistic vertebral trabecular attenuation measurements obtained from abdominal CT examinations for osteoporosis detection and to investigate their relationship with DXA-derived BMD parameters.

## METHODS

### Study design and patient population

This retrospective study was conducted between 2022 and 2025. The study was approved by the Clinical Research Ethics Committee of Cumhuriyet University (Decision No: 2025-12/26, Date: 11.12.2025) and was performed in accordance with the principles of the Declaration of Helsinki. Due to the retrospective design of the study, written informed consent was not obtained from individual patients. The study protocol and informed consent documentation were reviewed and approved by the ethics committee. All patient data were anonymized before analysis.

Abdominal CT examinations had originally been performed for routine non-oncologic clinical indications, including evaluation of abdominal pain, gastrointestinal disorders, hepatobiliary diseases, urinary tract disorders, and follow-up of benign abdominal conditions. Patients who underwent CT for oncologic staging, cancer surveillance, or major traumatic injuries were not included.

Eligible patients were consecutively identified through the institutional Picture Archiving and Communication System (PACS) and electronic medical record system by screening all patients who underwent both abdominal CT and DXA examinations between 2022 and 2025. All consecutive patients fulfilling the predefined inclusion and exclusion criteria were included, regardless of the clinical indication for DXA examination.

Patients who had both DXA and abdominal CT examinations performed within a maximum interval of 3 months were included in the study. A maximum interval of 3 months was considered acceptable, as previous opportunistic CT studies have accepted intervals of up to 6 months between CT and DXA examinations⁸. Therefore, the interval used in the present study was more stringent. Patients with systemic diseases known to affect bone metabolism (including chronic kidney disease, chronic liver disease, endocrine disorders affecting bone metabolism, inflammatory rheumatic diseases, and metabolic bone diseases other than primary osteoporosis), a history of malignancy, secondary causes of osteoporosis, vertebral fixation hardware, or vertebral compression fractures, scoliosis, severe degenerative changes, or other conditions affecting DXA interpretation were excluded. A history of malignancy and relevant comorbidities was verified using the institutional electronic medical record system. In addition, examinations with significant image-degrading artifacts and cases in which the interval between DXA and CT exceeded 3 months were also excluded.

A total of 451 patients were initially identified through the institutional PACS. After applying the predefined exclusion criteria, 15 patients with vertebral compression fractures, 12 with vertebral fixation hardware, 22 with solid malignancies, 12 with significant image artifacts, and 40 with an interval greater than 3 months between DXA and CT examinations were excluded. The final study population consisted of 350 patients.

Dual-energy X-ray absorptiometry and computed tomography evaluation

Bone mineral density measurements were performed using a Quantitative Digital Radiography (QDR) Hologic 4500W DXA system (Hologic Inc., Bedford, MA, USA), with patients examined in the supine position. Lumbar spine (L1–L4) T-scores were obtained from DXA scans. T-scores were calculated using the manufacturer-provided reference database of the Hologic DXA system. Lumbar spine assessment was performed using the L1–L4 region in all patients. Patients were classified according to the World Health Organization (WHO) criteria based on L1–L4 T-scores derived from DXA. Accordingly, patients with a T-score ≥-1 were defined as normal, those with T-scores between -1 and -2.5 were classified as osteopenia, and those with T-scores ≤-2.5 were classified as osteoporosis.

Computed tomography examinations were performed using a 128-detector CT scanner (GE Revolution EVO, Milwaukee, WI, USA) with a standardized abdominal CT protocol. Acquisition parameters included a tube voltage of 120 kVp, automated tube current modulation (100–250 mAs; target noise index, 14), a pitch of 0.984:1, and a gantry rotation time of 0.5 s. Axial datasets were acquired with 1.25-mm detector collimation and reconstructed at 1.0-mm slice thickness using a standard soft-tissue reconstruction kernel (Standard/Medium Smooth). Sagittal multiplanar reformatted images (0.625-mm thickness) were generated for trabecular bone attenuation measurements and analyzed using the Sectra PACS system (Sectra AB, Linköping, Sweden). Measurements were performed on the L1, L2, L3, and L4 vertebral bodies using equal-sized circular regions of interest (ROIs) with a fixed area of 100 mm^2^. For each vertebral body, HU measurements were obtained from a single mid-sagittal reconstructed image. To ensure accurate assessment of trabecular bone, the ROI was placed within the central trabecular portion of the vertebral body while avoiding cortical bone, end-plate regions, focal sclerotic or lytic lesions, and vertebral venous structures. All measurements were independently performed by two radiologists who were blinded to patient clinical data and to each other’s measurements. For statistical analysis, the mean values of measurements obtained by both observers were used (Figure 1).

**Figure 1:**
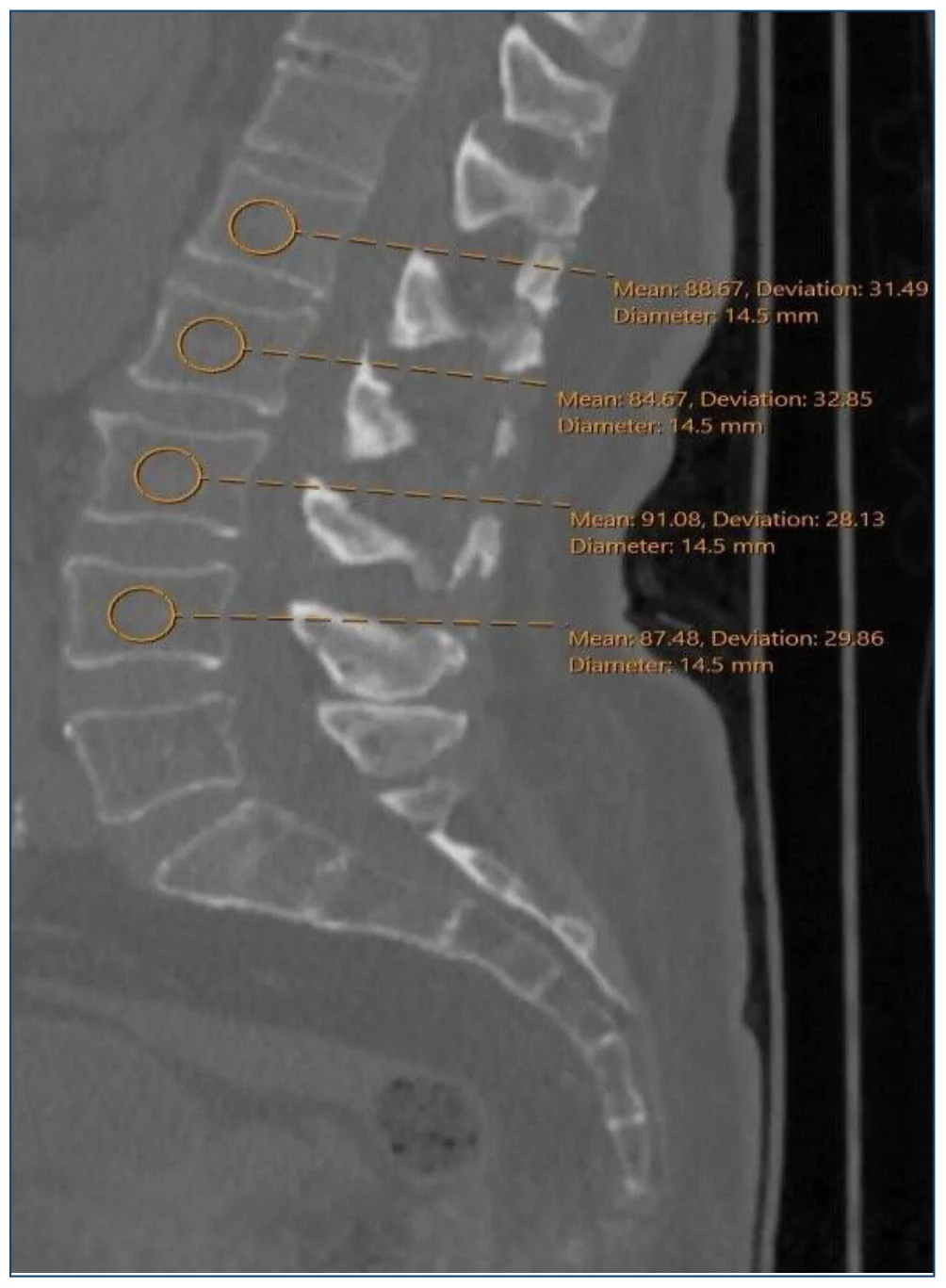
Sagittal reconstructed computed tomography image demonstrating the placement of regions of interest within the trabecular bone of L1–L4 vertebral bodies for Hounsfield Unit measurement.

### Statistical analysis

Statistical analyses were performed using Statistical Package for the Social Sciences (SPSS) software (Version 23.0; IBM Corp., Armonk, NY, USA). Continuous variables were expressed as mean±standard deviation and categorical variables as frequencies and percentages. Data normality was assessed using the Kolmogorov-Smirnov test. Comparisons among the normal, osteopenic, and osteoporotic groups were performed using one-way analysis of variance (ANOVA) for continuous variables and the chi-square test for categorical variables. Post-hoc pairwise comparisons were conducted using Tukey’s honest significant difference test.

Interobserver agreement for HU measurements was assessed using the intraclass correlation coefficient (ICC). Receiver operating characteristic (ROC) curve analysis was performed to evaluate the diagnostic performance of lumbar vertebral HU measurements for osteoporosis prediction, and optimal cutoff values were determined using the Youden index. A p<0.05 was considered statistically significant.

## RESULTS

A total of 350 patients were included in the study; 119 were classified as normal (34%), 117 as osteopenia (33.4%), and 114 as osteoporosis (32.6%). There were no statistically significant differences among the groups in terms of age, sex distribution, or body mass index (BMI) (all p>0.05). Lumbar vertebral HU measurements showed significant differences across all vertebral levels among the groups (p<0.001). Mean L1–L4 HU values demonstrated a progressively decreasing pattern from the normal group to the osteoporotic group, with the highest values observed in the normal group and the lowest in the osteoporosis group (148.99±5.54, 120.39±3.84, and 78.53±3.36, respectively; p<0.001) (Table 1).

**Table 1.** Baseline demographic characteristics and lumbar vertebral computed tomography attenuation values according to dual-energy X-ray absorptiometry -defined bone mineral density groups.

Variable	Normal (n=119)	Osteopenia (n=117)	Osteoporosis (n=114)	p-value
Age (years)	67.8±7.21	68.5±6.92	69.1±7.55	0.42
Female, n (%)	81 (68.1)	80 (68.4)	81 (71.1)	0.89
Male, n (%)	38 (31.9)	37 (31.6)	33 (28.9)	0.91
BMI (kg/m^2^)	33.51±2.43	29.03±0.64	28.80±1.03	0.07
L1 HU	154.21±5.85	128.94±3.98	76.56±4.14	**<0.001**
L2 HU	148.50±6.13	122.60±4.28	76.80±3.89	**<0.001**
L3 HU	145.71±6.09	111.93±3.89	78.04±3.77	**<0.001**
L4 HU	147.28±6.34	115.14±4.13	79.95±3.71	**<0.001**
Mean L1–L4 HU	148.99±5.54	120.39±3.84	78.53±3.36	**<0.001**

Continuous variables were compared using one-way analysis of variance (ANOVA), and categorical variables were compared using the chi-square test. Bold values indicate statistically significant results (p <0.05). BMI: body mass index; HU: Hounsfield Unit.

Mean L1–L4 HU values showed a significant difference among the groups (p<0.001).

According to Tukey’s post-hoc analysis, all pairwise comparisons among groups for L1–L4 vertebral HU values and mean L1–L4 HU values demonstrated statistically significant differences (all p<0.001). The normal group exhibited the highest HU values, whereas the lowest values were observed in the osteoporosis group. The osteopenia group showed intermediate HU values between the normal and osteoporosis groups.

ROC curve analysis demonstrated that lumbar vertebral HU measurements had significant diagnostic performance for predicting osteoporosis (all p<0.001) (Table 2). Among all vertebral levels, the highest diagnostic accuracy was observed for L1 HU measurements (area under the curve [AUC]: 0.913; 95%CI 0.870–0.955). An L1 cutoff value of ≤95.5 HU yielded a sensitivity of 84.6% and a specificity of 86.4% for the diagnosis of osteoporosis. Mean L1–L4 HU values also demonstrated high diagnostic performance (AUC: 0.881; 95%CI 0.835–0.928), with a cutoff value of ≤95 HU providing 80.8% sensitivity and 77.3% specificity. Although L3 and L4 HU measurements showed relatively lower diagnostic performance, all vertebral HU parameters were statistically significant in predicting osteoporosis.

**Table 2.** Diagnostic performance of lumbar vertebral Hounsfield Unit measurements for predicting osteoporosis: receiver operating characteristic curve analysis and optimal cutoff values.

	Cutoff (HU)	Sensitivity (%)	Specificity (%)	AUC	95%CI	p-value
L1 HU	**≤95.50**	84.60	86.40	0.913	0.870–0.955	**<0.001**
L2 HU	**≤92.50**	80.80	72.70	0.861	0.809–0.912	**<0.001**
L3 HU	**≤92.50**	73.10	70.50	0.819	0.759–0.878	**<0.001**
L4 HU	**≤96.50**	72.40	70.50	0.819	0.759–0.878	**<0.001**
Mean L1–L4 HU	**≤95.00**	80.80	77.30	0.881	0.835–0.928	**<0.001**

AUC: area under the receiver operating characteristic curve; CI: confidence interval; HU: Hounsfield Unit; AUC: area under the curve. Bold values indicate statistically significant results (p<0.05) and cutoff HU values.

To evaluate the potential confounding effect of BMI, multivariable logistic regression analyses were performed for each vertebral HU parameter (L1–L4 and mean L1–L4). After adjustment for BMI, all vertebral HU measurements remained independently associated with osteoporosis (L1: OR 0.935, 95%CI 0.913–0.958; L2: OR 0.955, 95%CI 0.940–0.971; L3: OR 0.961, 95%CI 0.947–0.975; L4: OR 0.964, 95%CI 0.950–0.977; mean L1–L4: OR 0.941, 95%CI 0.921–0.961; all p<0.001), whereas BMI was not an independent predictor in any of the models (all p>0.05).

Interobserver agreement for lumbar vertebral HU measurements demonstrated excellent reliability between the two radiologists, with ICC values ranging from 0.90 to 0.94.

## DISCUSSION

In this study, we evaluated the diagnostic value of opportunistic vertebral trabecular attenuation measurements obtained from unenhanced abdominal CT scans in identifying osteoporosis according to DXA-based WHO criteria. Lumbar vertebral HU values at all levels (L1–L4) and mean L1–L4 HU values showed a significant stepwise decrease across normal, osteopenic, and osteoporotic groups (p<0.001). Furthermore, excellent interobserver agreement (ICC: 0.90–0.94) demonstrates the high interobserver reliability of this method, supporting its use as an objective opportunistic screening tool.

Previous studies have demonstrated that vertebral HU measurements derived from routine abdominal and thoracic CT examinations can be used for opportunistic osteoporosis screening without additional radiation exposure or additional imaging. Pickhardt et al.^8^, in a cohort of 1,867 patients, reported a strong correlation between L1 HU values and DXA T-scores, with high sensitivity for osteoporosis detection. Similarly, Pickhardt et al.^7^ showed that L1 trabecular attenuation measurements obtained from CT colonography achieved sensitivities approaching 100% for identifying low BMD. These findings are consistent with the progressive decline in HU values and the high diagnostic performance observed in our study.

ROC analysis demonstrated that the highest diagnostic accuracy for osteoporosis prediction was achieved at the L1 vertebral level (AUC: 0.913). An L1 cutoff value of ≤95.5 HU yielded 84.60% sensitivity and 86.40% specificity. Zhu et al.^14^ reported that optimal L1 thresholds generally range from 110 to 135 HU, whereas values ≤100 HU are highly specific for osteoporosis. Therefore, the cutoff identified in our study is consistent with the lower threshold range reported in the literature. Differences among studies may be related to variations in patient demographics and CT acquisition parameters. Lee et al.^15^ further demonstrated that every 10-HU decrease in L1 trabecular attenuation is associated with a significantly increased risk of future osteoporotic fractures, highlighting the clinical value of identifying patients with low HU values for earlier DXA evaluation and treatment. The superior diagnostic performance of L1 compared with the mean L1–L4 attenuation may reflect vertebral-level heterogeneity, which could reduce the diagnostic performance of averaged measurements. The optimal cutoff values were determined using the Youden index; however, alternative thresholds may be preferred depending on the intended clinical application.

A major strength of our study is the use of sagittal reconstructed images for HU measurements. Although most opportunistic screening studies have traditionally relied on axial images, recent investigations have compared different imaging planes. Kim et al.^16^ reported no significant differences in diagnostic performance among coronal, axial, and sagittal planes, with excellent interobserver agreement for all approaches (ICC>0.90). The authors also emphasized that sagittal and coronal reconstructions may facilitate exclusion of degenerative changes such as osteophytes and end-plate sclerosis while enabling more accurate ROI placement. Consistent with these findings, the sagittal approach used in our study minimized degenerative interference and demonstrated excellent reliability (ICC: 0.90–0.94). Although our findings are generally consistent with previous opportunistic CT studies, the present study adds to the existing literature by demonstrating the diagnostic performance and excellent interobserver reliability of sagittal HU measurements in a Turkish population. These findings support the reproducibility and potential clinical applicability of sagittal plane assessment in routine abdominal CT examinations.

Conventional DXA may overestimate BMD in older individuals because of aortic calcifications, osteophytes, and facet joint degeneration^7,14^. In contrast, CT-based ROI placement within trabecular bone reduces the impact of these artifacts. The marked decline in HU values across the normal, osteopenic, and osteoporotic groups (148.99, 120.39, and 78.53 HU, respectively) indicates that CT attenuation measurements closely reflect DXA-based classifications while providing a more objective assessment.

Although the normal group had a higher mean BMI than the osteopenia and osteoporosis groups, multivariable logistic regression analyses demonstrated that BMI was not an independent predictor of osteoporosis after adjustment for vertebral HU measurements. In contrast, all vertebral HU parameters remained independently associated with osteoporosis. These findings suggest that the diagnostic performance of vertebral HU measurements was not substantially influenced by differences in BMI.

Although the present study demonstrates the diagnostic potential of opportunistic CT-based HU measurements, practical implementation in routine clinical practice will require standardized measurement protocols and, ideally, automated software tools to facilitate workflow integration. In clinical practice, patients with markedly reduced vertebral attenuation values, such as L1 HU values around or below the threshold identified in this study, may be considered for DXA referral based on individual risk factors. Further prospective studies are needed to evaluate implementation feasibility and clinical impact. In addition, the proposed cutoff values should be interpreted in the context of opportunistic screening rather than definitive diagnosis, and positive findings should be confirmed by DXA. Integration of opportunistic bone assessment with other CT-based screening approaches may represent a potential future strategy to maximize the clinical value of routinely acquired CT examinations. Future studies should evaluate automated HU measurement approaches, prospective validation of cutoff values, cost-effectiveness, and integration with electronic health record systems to facilitate broader clinical implementation. From a health economic perspective, opportunistic CT-based HU assessment may reduce healthcare costs by utilizing existing imaging data without additional examinations.

Several limitations should be acknowledged. First, the retrospective single-center design and inclusion of patients who underwent DXA as part of routine clinical care may limit generalizability and introduce selection bias, although consecutive patient inclusion was applied to minimize this effect. Second, intravenous contrast administration may artificially increase vertebral HU values by approximately 10–30 HU^8,16^. To avoid this effect, only non-contrast CT examinations were included; therefore, our findings are directly applicable only to non-contrast CT examinations. Although many abdominal CT studies in routine practice are contrast-enhanced, correction methods have been proposed for contrast-enhanced CT, but these have not yet been standardized. Future studies incorporating standardized correction factors may improve applicability in these settings. In addition, the proposed HU cutoff values were not internally validated using cross-validation or bootstrapping. Therefore, external validation in independent cohorts is warranted before widespread clinical implementation. Furthermore, although excellent interobserver agreement was demonstrated, intraobserver variability was not assessed. Finally, long-term fracture follow-up data were unavailable.

## CONCLUSION

Opportunistic sagittal lumbar vertebral HU measurements obtained from routine abdominal CT scans, particularly at the L1 level and using mean L1–L4 values, provide a reliable, highly reproducible method for opportunistic osteoporosis screening without requiring additional imaging or radiation exposure. Integration of ROI-based HU assessment into routine clinical workflows may facilitate earlier diagnosis and support timely fracture-prevention strategies.

## Data Availability

The datasets generated and/or analyzed during the current study are available from the corresponding author upon reasonable request.
